# Close, but no cigar: an unfortunate case of primary angiitis of the central nervous system

**DOI:** 10.1097/MS9.0000000000000084

**Published:** 2023-02-17

**Authors:** Branden Ireifej, Julie Kanevsky, David Song, Talal Almas, Abdulla K. Alsubai, Sebastian Hadeed, Khaled S.O. Aldhaheri, Hussein Khan Ali, Helen Huang, Olivia Ghaw

**Affiliations:** a Department of Internal Medicine, Icahn School of Medicine at Mount Sinai, Elmhurst Hospital Center, Queens; b Department of Rheumatology, Icahn School of Medicine at Mount Sinai Hospital, New York, New York, USA; c Royal College of Surgeons in Ireland, Dublin, Ireland

**Keywords:** Primary angiitis, CNS, Nervous system, Internal medicine, Medicine

## Abstract

Primary angiitis of the central nervous system (PACNS) is an uncommon and misunderstood disease, where little is known regarding its immunopathogenesis and appropriate treatment. Due to the constellation of nonspecific clinical features and imaging findings, PACNS is a diagnostic conundrum for clinicians and can be very difficult to treat.

**Case Presentation:**

A 64-year-old male with a history of prostate cancer presented to the emergency department with expressive aphasia and severe headache. Previously, he was diagnosed with ischemic strokes at outside hospitals and was subsequently initiated on anticoagulation medication but was later readmitted with a new onset of nontraumatic subarachnoid hemorrhage and later was found to have ischemic changes in the right temporoparietal lobe. He was suspected to have hypercoagulability of malignancy, as he was unresponsive to a wide variety of anticoagulants and his symptoms continued to deteriorate. On presentation, the physical examination was significant for right homonymous hemianopia, with positive antinuclear antibodies and notable erythrocyte sedimentation rate. The results from the full serologic workup was negative. Subsequent imaging of the brain revealed multifocal stenoses in multiple arteries. On further examination, digital subtraction angiography was concerning for vasculopathy, and was initiated on corticosteroids and cyclophosphamide.

**Discussion:**

This is one of the first cases of PACNS in which recurrent strokes were the presenting symptom for PACNS. Vasculitis should be a considered differential in patients with recurrent ischemic strokes and failed anticoagulant therapy. It is important to rule out malignancy and infectious causes due to the wide spectrum of conditions that cause central nervous system vasculitis.

Key points

- Primary angiitis of the central nervous system (PACNS) is an uncommon and misunderstood disease, where little is known regarding its immunopathogenesis and appropriate treatment.
- This is one of the first cases of PACNS in which recurrent strokes were the presenting symptom for PACNS.
- Vasculitis should be a considered differential in patients with recurrent ischemic strokes and failed anticoagulant therapy

PACNS is a very rare form of vasculitis characterized by central nervous system (CNS) inflammation and cerebral ischemia with no secondary cause^1^. With a prevalence of 2.4 cases per 1 000 000 patients, PACNS is uncommon and a poorly understood disease with little information regarding its immunopathogenesis and etiologies^2^. The condition was first discovered as its own clinical condition in 1959 and has since emerged with a wide range of nonspecific clinical presentations, causing a diagnostic conundrum for clinicians in the modern world^3^. The most frequently reported symptom of PACNS were headaches that are progressive in nature, but often do not signal an evaluation for risk of subarachnoid hemorrhages^4^. Cognitive changes and focal neurological deficits such as aphasia or hemiparesis have also been frequently described features, while strokes and transient ischemic attacks have been reported in 30–50% of patients^5^,^6^. The clinical signs and symptoms of PACNS differ on an individual case-by-case basis, and imaging findings tend to not be specific. The lack of studies on PACNS coupled with an absent universal diagnostic criterion makes this condition extremely difficult to treat and can be detrimental if undiagnosed. We describe a patient who presented with multiple recurrent ischemic strokes despite adherence to anticoagulation therapy and was later diagnosed with PACNS upon extensive investigations. The case highlights a rare presentation of PACNS and recognizes the need to initiate prompt treatment.

## Case presentation

A 64-year-old male presented to the emergency department with expressive aphasia and severe diffuse headache. He had a history of hypertension, prostate cancer, and lifelong smoking. Over the previous 6 months the patient had recurrent transient episodes of weakness and aphasia for which he was evaluated at an outside hospital. His first hospital presentation showed a multifocal stroke on MRI, where he was discharged on rivaroxaban. However, he was readmitted 1 month later with worsening residual weakness, presumably caused by anticoagulation failure. Repeat imaging showed a new onset of right frontal nontraumatic subarachnoid hemorrhage along with ischemic changes in the right temporoparietal lobe. Rivaroxaban was changed to dual antiplatelet therapy, but the patient returned with persistent symptoms a week after discharge. An MRI showed evolving ischemic changes in the same area. Echocardiography and lumbar puncture were unremarkable. Apixaban was started despite subarachnoid hemorrhage given the suspicion for evolving embolic phenomena. Two months later, the patient had a follow-up MRI demonstrating a new right occipital stroke and diffuse cervical spine metastases. Hypercoagulability of malignancy was suspected, prompting a switch from apixaban to enoxaparin.

**Figure 1:**
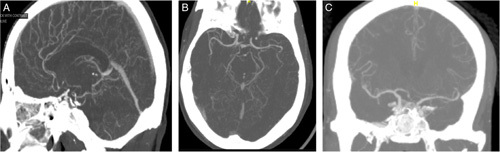
(A–C) Computed tomography angiogram of the brain showed multifocal stenosis in anterior cerebral artery, posterior cerebral artery, and middle cerebral artery territories.

**Figure 2:**
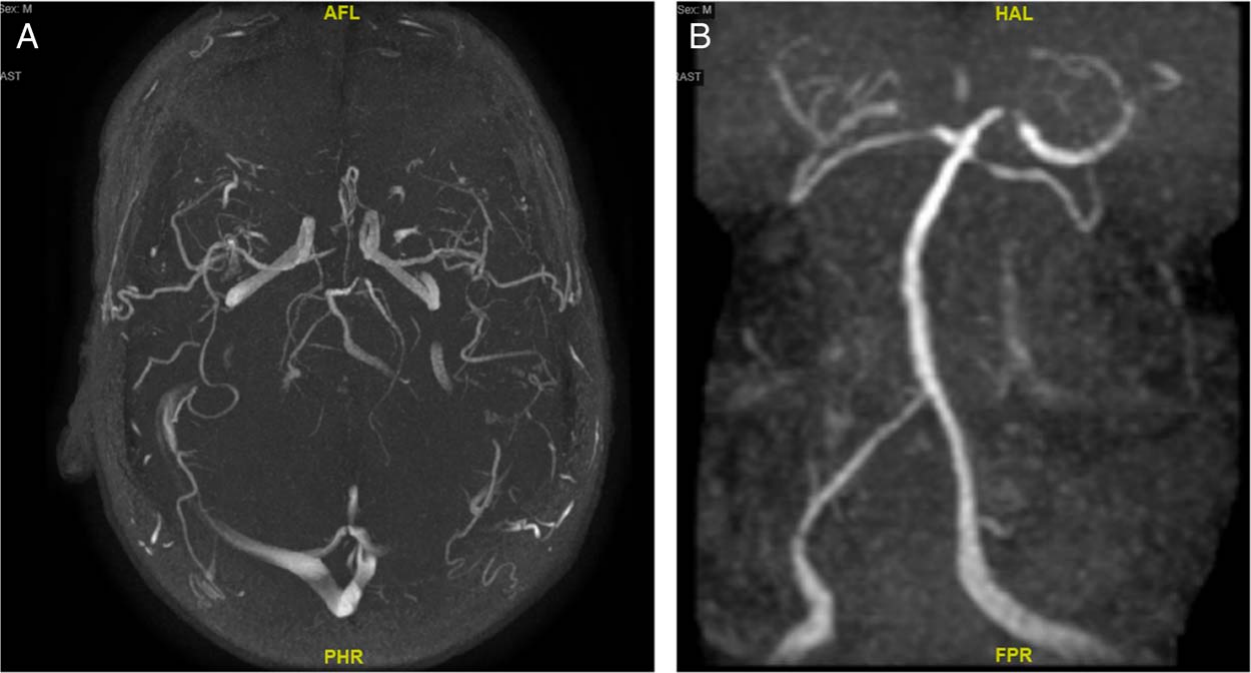
(A and B) Magnetic resonance angiogram of the brain demonstrated multifocal severe stenosis of the anterior cerebral artery, middle cerebral artery, posterior cerebral artery, and superior cerebellar artery.

Based off his current presentations, vital signs were unremarkable, and the physical examination was significant for right homonymous hemianopia. Labs were notable for erythrocyte sedimentation rate of 41 mm/h and positive antinuclear antibodies (1:160; speckled). Complete blood count, complete metabolic panel, and infectious workup were unremarkable. Serologic workup, including antineutrophil cytoplasmic antibody panel, lupus anticoagulant antibody, Sjogren’s antibody, anti–double-stranded DNA, homocysteine antibody, and anticardiolipin antibody were all negative as well. Vasculitis was suspected, prompting the administration of intravenous methylprednisolone 1 g daily for 3 days followed by prednisone 60 mg daily. A computed tomography angiogram of the brain was ordered and showed multifocal stenosis in anterior cerebral artery, posterior cerebral artery, and middle cerebral artery territories (Fig. 1A–C). A magnetic resonance angiogram of the brain demonstrated multifocal severe stenosis of the anterior cerebral artery, middle cerebral artery, posterior cerebral artery, and superior cerebral artery (Fig. 2A and B). The patient underwent digital subtraction angiography which showed vasculopathy. Brain biopsy was inconclusive. Based on our findings, he was diagnosed with PACNS. He was continued on corticosteroids and started on cyclophosphamide 1 g per month induction therapy. However, the patient continued to have recurrent in-hospital strokes while on combination immunosuppressants. Unfortunately, he passed away on hospital day 30.

## Discussion

PACNS is a poorly understood disease in which the CNS is the sole or dominant target organ of a vasculitic process, affecting the leptomeningeal and cortical arteries^7^. Vasculitis should be considered in patients with recurrent ischemic strokes and multiple failed anticoagulant treatment. The spectrum of diseases that can cause vasculitis of the CNS can be broad, making it important to rule out malignancy and infectious causes. In our patient, thorough investigations deemed the patient to have no other source of the vasculitis, ultimately determining it to be primary angiitis. Diagnostic workup includes imaging, laboratory workup, and a potential brain biopsy if imaging is equivocal. Our patient’s brain biopsy was inconclusive, but it is important to note that he was initiated on high-dose steroids well before the procedure. Furthermore, due to the focal and segmental distribution of the disease, the sensitivity of the brain biopsy is 53–74%^8^,^9^. Nonetheless, a negative biopsy does not rule out PACNS, but rather provide histopathologic results that offer an alternative to diagnosing the disease, in which case showed vasculopathy^10^. Subsequent contrast tomography angiogram and magnetic resonance angiogram were also highly characteristic of vasculitis.

PACNS generally presents in the fourth decade of life with headaches, altered cognition, and focal neurological deficits^11^,^12^. However, there have been reported cases of recurrent strokes secondary to vasculitis of the CNS as the initial presentation^7^,^11^. Treatment recommendations for PACNS are mainly based on retrospective studies and expert opinions. However, therapy of PACNS with a combination of cytotoxic drugs and high-dose corticosteroids has greatly improved the prognosis for this condition^4^.

In our patient, his recurrent strokes at outside institutions were unfortunately deemed to be a component of hypercoagulability of malignancy from his prostate cancer. This inflammatory disease can vary greatly in presentation, and little is known regarding its pathogenesis nor suitable treatment options. The majority of data for this disease is derived from observational studies.

## Conclusion

PACNS is rare but can prove life-threatening. Prompt recognition of symptoms is important as treatment can potentially alter morbidity.

## Limitations

A limitation from this case study is a lack of clinical information in the literature regarding PACNS. Because this was one of the first cases of PACNS presenting with recurrent strokes, there is no information in databases that could serve as a comparison. Thus, information on the condition should be taken from this case with caution.

## Ethical approval

Not applicable.

## Sources of funding

None.

## Authors’ contribution

B.I. and J.K.: conceived the idea, designed the study, and drafted the manuscript. D.S. and O.G.: conducted comprehensive literature search, screened the studies for relevant content, and created the literature review table. T.A. and A.K.A.: revised the manuscript critically and refined the literature review table. S.H. and H.K.A.: drafted the discussion part of the manuscript, revised the final version of the manuscript critically based on the reviewer and editorial comments. H.H.: conceived the initial study idea, diagnosed the case, and gave the final approval for publication.

## Conflicts of interest disclosure

The authors declare that they have no financial conflict of interest with regard to the content of this report.

## Research registration unique identifying number (UIN)

Not applicable.

## Guarantor

Talal Almas.

## Consent

Written informed consent was obtained from the patient for publication of this case report and accompanying images. A copy of the written consent is available for review by the Editor-in-Chief of this journal on request.
